# Efficacy of orthodontic and orthognathic treatment for oral and maxillofacial deformities

**DOI:** 10.1097/MD.0000000000017324

**Published:** 2019-09-27

**Authors:** Jin-Yu Gao, Xiao-Qin Yu

**Affiliations:** a Department of Orthodontics, Yan’an University Affiliated Stomatological Hospital, Yan’an; b Department of Stomatology, Ankang People's Hospital, Ankang, China.

**Keywords:** efficacy, oral and maxillofacial deformities, orthodontic and orthognathic treatment, safety

## Abstract

**Background:**

This study aims to assess the efficacy and safety of orthodontic and orthognathic treatment (OOT) for patients with oral and maxillofacial deformities (OMDF) systematically.

**Methods:**

This study will comprehensively search Cochrane Library, PubMed, EMBASE, Scopus, Web of Science, PsycINFO, Index to Nursing and Allied Health Literature, Allied and Complementary Medicine Database, Chinese Biomedical Literature Database, and China National Knowledge Infrastructure from their inceptions to the July 1, 2019. Grey literature will be explored via searching dissertations, Google scholar and conference abstracts. Two team members will independently perform all citations, data extraction, and methodological quality. We will also utilize RevMan 5.3 Software for statistical analysis.

**Results:**

This study will provide high quality evidence of OOT for OMDF. The primary outcomes consist of number of patients cured; proportion of patients healed; and time to complete healing within trial period. Secondary outcomes include quality of life (often assessed as any relevant scales, such as 36-Item Short Form Survey), costs, and complications.

**Conclusion:**

This study will provide evidence for judging whether OOT is effective treatment for OMDF.

**Systematic review registration:**

CRD42019144610.

## 1 Introduction

Oral and maxillofacial deformities (OMDF) are characterized as irregularities or malformations in the bones and/or soft tissues of the mouth, jaws, and face.^[1–4]^ Several factors can cause such disease, including cleft lip, cleft palate, cancer, trauma, and automobile accident.^[5–11]^ It has been reported that about 20% of the world's population have some major OMDF.^[12,13]^ It has greatly affected the health-related quality of life in patients with OMDF.^[14–16]^ The severity of patients with OMDF is very high which affects their facial proportion, and about 5% patients have physical disability.^[12]^

Surgery is a very effective treatment for patients with OMDF.^[2,4,5,7,8,17–23]^ Although a variety of studies have reported the efficacy and safety of surgery, especially for orthodontic and orthognathic treatment (OOT) for the management of OMDF, there is still inconsistent conclusion.^[2,4,18–22]^ Thus, this study will assess the efficacy and safety of OOT for the treatment of patients with OMDF.

## 2 Methods and analysis

### 2.1 Inclusion criteria for study selection

#### 2.1.1 Types of studies

Only randomized controlled trials (RCTs) of OOT for patients with OMDF will be included. However, non-clinical studies, and non-RCTs will be excluded.

#### 2.1.2 Types of participants

Patients with a clinically confirmed diagnosis of OMDF will be considered for inclusion regardless the race, gender, age, or economic status.

#### 2.1.3 Types of interventions

In the experimental group, all patients must receive OOT.

In the control group, all patients can receive any treatments, except OOT.

#### 2.1.4 Type of outcome measurements

Primary outcomes consist of number of patients cured; proportion of patients healed; and time to complete healing within trial period.

Secondary outcomes comprise of quality of life (often assessed as any relevant scales, such as 36-Item Short Form Survey), costs, and complications.

### 2.2 Literature search

We will search Cochrane Library, PubMed, EMBASE, Scopus, Web of Science, PsycINFO, Index to Nursing and Allied Health Literature, Allied and Complementary Medicine Database, Chinese Biomedical Literature Database, and China National Knowledge Infrastructure from their inceptions to the July 1, 2019. We will search all electronic databases without language restrictions. We will build search strategy sample for Cochrane Library in Table 1. We will also adapt similar search strategy to any other electronic databases. In addition, we will also search Grey literature, such as dissertations, Google scholar, and conference abstracts to avoid missing any potential studies.

**Table 1:**
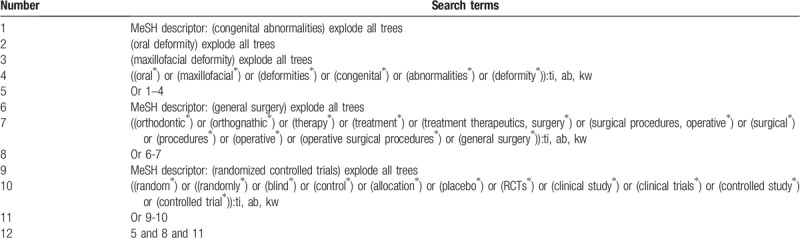
Search strategy applied in Cochrane Library database.

### 2.3 Study selection and extraction

#### 2.3.1 Study selection

Study selection will be performed by 2 independent authors, respectively. All disagreements between 2 authors will be solved by another author through discussion. The whole process consists of 2 steps. First, all records citation will be scanned using titles and abstracts, and all duplicated and irrelevant studies will be excluded. Second, all remaining studies will be further identified whether they meet all eligibility criteria. The excluded reasons for all literatures will be recorded at each step. The results of study selection will be presented in the flowchart of this study.

#### 2.3.2 Data extraction and management

All data extraction will be carried out by 2 independent authors. A third author will help to solve any divergences between 2 authors. All searched literature citations will be imported and managed using Endnote X7. A standard pre-designed sheet for data extraction will be built before the data collection. All essential information will be extracted, including title, first author, country, year of publication, study setting, study design, study methods, treatment details, outcome measurements, safety, and all other related information.

### 2.4 Risk of bias assessment

The risk of bias for each eligible study will be assessed independently by 2 authors according to the standard of Cochrane risk of bias tool, which focuses on 7 domains. Any disagreements between 2 authors will be solved by a third author via discussion to reach consensus.

### 2.5 Outcome measurement effects

Categorical outcome data will be calculated with risk ratio and 95% confidence intervals (CIs), while continuous outcome data will be expressed with mean difference or standardized mean difference and 95% CIs.

### 2.6 Heterogeneity assessment

We will utilize *I*^*2*^ test to assess heterogeneity among eligible studies. If *I*^*2*^ ≤ 50%, the heterogeneity is considered as homogeneity, and a fixed-effect model will be used. If *I*^*2*^ > 50%, the heterogeneity is considered as obvious, and a random-effect model will be utilized.

### 2.7 Statistical analysis

We will apply RevMan 5.3 software for statistical analysis. If homogeneity is identified (*I*^*2*^ ≤ 50%), then we will pool the data and carry out meta-analysis. If heterogeneity is obvious (*I*^*2*^ > 50%), we will perform subgroup analysis and meta-regression analysis to determine their potential sources. If there is still obvious heterogeneity, we will not pool the data, and will report outcome results as narrative summary.

### 2.8 Additional analysis

#### 2.8.1 Subgroup analysis

We will conduct subgroup analysis according to different treatments, and outcome measurements.

#### 2.8.2 Sensitivity analysis

We will perform sensitivity analysis by eliminating the high risk of bias studies.

#### 2.8.3 Reporting bias

We will carry out funnel plots, and Egger regression test^[24]^ to identify any potential reporting bias if sufficient studies are included.

### 2.9 Ethics and Dissemination

This study will not need ethical approval, because no individual patient data will be analyzed. This study will be disseminated through peer-reviewed journals.

## 3 Discussion

OMDF is a common disorder. As an effective treatment, OOT has been proved to enhance the health-related quality of life in patients with OMDF. However, there is still insufficient evidence to support the efficacy of OOT for OMDF, and no study has addressed its efficacy and safety for OMDF. Therefore, it is very necessary to conduct this study to assess the efficacy and safety of OOT for patients with OMDF. This study will provide a detailed summary of the present evidences related to the efficacy and safety of OOT for the treatment of OMDF. Its findings may be useful to patients, clinicians, and health policy-makers with regard to utilization of OMDF for patients with OOT.

## Author contributions

**Conceptualization:** Jin-yu Gao, Xiao-qin Yu.

**Data curation:** Jin-yu Gao, Xiao-qin Yu.

**Formal analysis:** Jin-yu Gao.

**Investigation:** Xiao-qin Yu.

**Methodology:** Jin-yu Gao.

**Project administration:** Xiao-qin Yu.

**Resources:** Jin-yu Gao.

**Software:** Jin-yu Gao.

**Supervision:** Xiao-qin Yu.

**Validation:** Jin-yu Gao, Xiao-qin Yu.

**Visualization:** Jin-yu Gao, Xiao-qin Yu.

**Writing – original draft:** Jin-yu Gao, Xiao-qin Yu.

**Writing – review & editing:** Jin-yu Gao, Xiao-qin Yu.
